# MCAF-Net: Multi-Channel Temporal Cross-Attention Network with Dynamic Gating for Sleep Stage Classification

**DOI:** 10.3390/s25144251

**Published:** 2025-07-08

**Authors:** Xuegang Xu, Quan Wang, Changyuan Wang, Yaxin Zhang

**Affiliations:** School of Computer Science and Engineering, Xi’an Technological University, Xi’an 710021, China; xuxuegang@st.xatu.edu.cn (X.X.); wangchangyuan@xatu.edu.cn (C.W.); zhangyaxin@st.xatu.edu.cn (Y.Z.)

**Keywords:** automatic sleep stage classification, multi-channel signal fusion, temporal convolution, dynamic gated, multi-head cross-channel attention mechanism

## Abstract

Automated sleep stage classification is essential for objective sleep evaluation and clinical diagnosis. While numerous algorithms have been developed, the predominant existing methods utilize single-channel electroencephalogram (EEG) signals, neglecting the complementary physiological information available from other channels. Standard polysomnography (PSG) recordings capture multiple concurrent biosignals, where sophisticated integration of these multi-channel data represents a critical factor for enhanced classification accuracy. Conventional multi-channel fusion techniques typically employ elementary concatenation approaches that insufficiently model the intricate cross-channel correlations, consequently limiting classification performance. To overcome these shortcomings, we present MCAF-Net, a novel network architecture that employs temporal convolution modules to extract channel-specific features from each input signal and introduces a dynamic gated multi-head cross-channel attention mechanism (MCAF) to effectively model the interdependencies between different physiological channels. Experimental results show that our proposed method successfully integrates information from multiple channels, achieving significant improvements in sleep stage classification compared to the vast majority of existing methods.

## 1. Introduction

Sleep is a fundamental pillar of human health, exerting profound influence on physical well-being, cognitive performance, and emotional stability [1]. Sleep deprivation or disruption is closely associated with a range of serious health conditions, including cardiovascular diseases, neurodegenerative disorders, and mental health issues [2]. Accurate sleep staging is essential for the diagnosis of sleep disorders and the assessment of sleep quality [3]. Clinically, polysomnography (PSG) remains the gold standard for sleep analysis, as it simultaneously records multiple physiological signals, such as electroencephalogram (EEG), electrooculogram (EOG), and electromyogram (EMG) [4]. According to the guidelines established by the American Academy of Sleep Medicine (AASM) [5,6], PSG signals are segmented into 30 s epochs and classified into five distinct stages: wakefulness (W), non-rapid eye movement (N1, N2, N3), and rapid eye movement (REM) sleep.

Historically, sleep staging relied on manual scoring by experts, a labor-intensive and subjective process prone to inefficiencies [7]. To address these limitations, automated sleep staging systems leveraging machine learning and deep learning have gained traction. Early approaches used traditional machine learning algorithms, such as k-nearest neighbor [8], support vector machines [9,10], and random forests [11,12], which required manual feature extraction [13]. However, these methods were heavily dependent on domain expertise, and the advent of deep learning has revolutionized sleep staging by enabling end-to-end feature learning. Convolutional neural networks (CNNs) have been widely adopted for their ability to extract spatial features from raw or time-frequency representations of PSG signals. For instance, Sors et al. [14] proposed a 14-layer CNN that processes a sequence of sleep epochs, leveraging contextual information from adjacent epochs to enhance stage classification. Similarly, recurrent neural networks (RNNs), such as SeqSleepNet [15], employ bidirectional RNNs to model temporal dependencies across sleep epochs, improving sequence-level analysis. Despite their strengths, CNNs often fail to capture temporal correlations, while RNNs face challenges with high computational complexity and limited parallelization. To address these limitations, hybrid models combining CNNs and RNNs have gained popularity. For example, DeepSleepNet [16] integrates CNNs with varied kernel sizes to extract features from single-channel EEG epochs, followed by a long short-term memory (LSTM) network to capture transitions between sleep stages. Likewise, SleepEEGNet [17] uses CNNs for time-invariant feature extraction and bidirectional RNNs to model contextual relationships between epochs. Additionally, Korkalainen et al. [18] combined convolutional and LSTM networks to investigate the impact of obstructive sleep apnea severity on staging accuracy, highlighting the potential of hybrid architectures for specific clinical applications.

Inspired by Transformer networks, recent studies have adopted attention mechanisms to accomplish the task of sleep stage classification. Eldele et al. [19] introduced a temporal context encoder leveraging causal convolution-enhanced multi-head self-attention for temporal dependency capture. SleepTransformer [20] employs a transformer backbone to model intra-epoch and inter-epoch dependencies, offering interpretable predictions but with moderate performance. Multi-channel approaches have also emerged to leverage the complementary information in PSG signals. Jia et al. [21] introduced a squeeze-and-excitation-based fusion method to integrate EEG and EOG features, though it risked losing local spatial information during global pooling. Similarly, MultiChannelSleepNet [22] uses transformer encoders for single-channel feature extraction and multi-channel fusion, but its concatenation-based fusion may underutilize inter-channel relationships.

While multi-channel PSG signals offer rich information, effective fusion of these signals remains a challenge. Simple concatenation or pooling strategies often fail to capture dynamic inter-channel interactions, and complex models can introduce computational overhead unsuitable for resource-constrained applications. To address these gaps, we propose MCAF-Net, a lightweight Multi-Channel Temporal Cross-Attention Network with Dynamic Gating for sleep stage classification. MCAF-Net processes time-frequency representations of EEG and EOG signals, employing a temporal convolution module to extract channel-specific features. A novel Channel-Aware Attention mechanism facilitates dynamic cross-channel feature fusion, enhanced by a gating mechanism that adaptively modulates feature contributions. This design ensures efficient information integration while maintaining a compact model architecture.

Our main contributions are as follows:

We introduce a lightweight temporal convolution module that independently extracts temporal features from each PSG channel, preserving modality-specific information.

We propose a Channel-Aware Attention module with dynamic gating, enabling adaptive and effective fusion of multi-channel features by modeling cross-channel interactions.

MCAF-Net achieves superior performance on benchmark datasets (e.g., Sleep-EDF-20 and Sleep-EDF-78) while maintaining low computational complexity, making it suitable for real-world sleep monitoring applications.

## 2. Methodology

**Figure 1:**
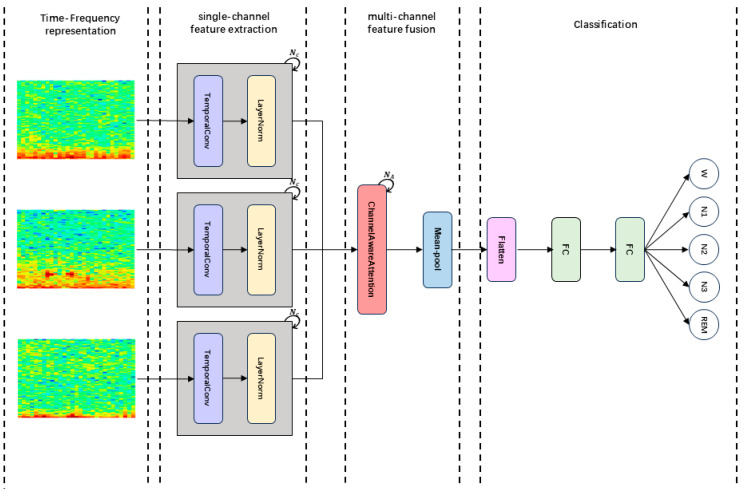
The overall architecture of MCAF-Net: (1) Constructing time-frequency representations of multi-channel signals. (2) Extracting features from each channel individually. (3) Integrating multi-channel features through a fusion block. (4) Performing classification via two fully connected layers.

In this section, we present our model, MCAF-Net. As shown in Figure 1, it mainly consists of four modules: Time-Frequency representation, TemporalConv for single-channel feature extraction, MCAF for multi-channel feature fusion, and Classification.

### 2.1. Time-Frequency Representation

According to the AASM scoring manual [5], EEG, EOG, EMG, and major body movements serve as the basis for sleep staging, with specific waves and frequency components being critical features. For instance, low-frequency components in the 4–7 Hz range are frequently observed during the N1 stage, while sleep spindles (SS) or K-complexes (KC) are hallmark features of the N2 stage. Slow waves predominantly appear in the N3 stage. By applying the Short-Time Fourier Transform (STFT) and logarithmic scaling, we convert the raw signals from each channel into time-frequency images as model inputs, which effectively represent these specific waves and frequency components, thereby improving the accuracy of sleep staging.

### 2.2. Single-Channel Feature Extraction

Sleep represents a continuous physiological process in which transitions between distinct sleep stages exhibit intrinsic interdependencies rather than occurring in isolation. Consequently, the comprehension and modeling of such temporal dynamics are of paramount importance for sleep staging tasks.

**Figure 2:**
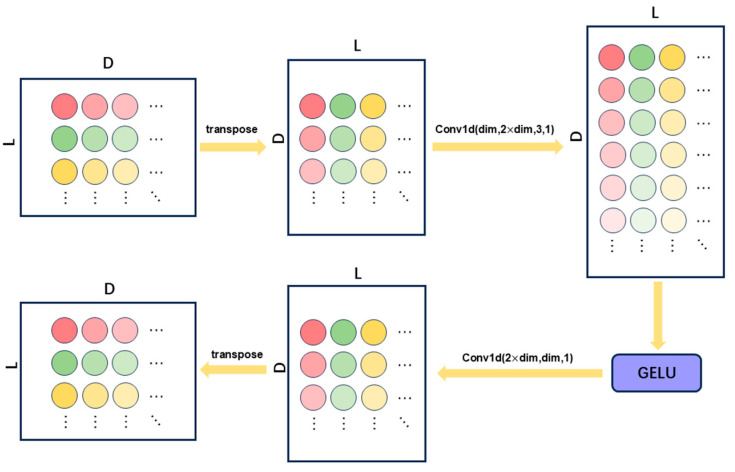
Structure of TemporalConv.

To effectively extract temporal features from time-frequency representations across individual channels, we propose a dedicated single-channel feature extraction module termed TemporalConv, designed to process each channel’s temporal data independently. As illustrated in Figure 2, the core architecture of TemporalConv comprises two convolutional layers, augmented with nonlinear activation functions and transposition operations to facilitate temporal feature extraction. This module demonstrates both high efficiency in capturing channel-wise temporal patterns and low computational complexity.

The input tensor is first transposed from [B,L,D] to [B,D,L] (where B denotes the batch size, L represents the sequence length, and D indicates the number of feature channels per time step) to accommodate the input format of 1D convolutional operations. A 1D convolutional layer is then applied independently to each channel, using a kernel size of 3 and padding of 1 to preserve the original temporal sequence length. The number of output channels is expanded to 2×D to enhance feature representation capacity.

Nonlinearity is introduced via the GELU activation function, improving the model’s ability to capture complex temporal patterns. A 1×1 convolutional layer subsequently reduces the feature dimension from 2×D back to D, achieving both feature compression and parameter efficiency. Finally, the tensor is transposed back to its original [B,L,D] configuration for seamless integration with subsequent processing modules.

### 2.3. Multi-Channel Feature Fusion

Following single-channel feature extraction, MCAF-Net employs a multi-channel feature fusion module to enable inter-channel information interaction and integration. The inherent multi-channel characteristics of sleep signals make cross-channel feature fusion particularly essential, as temporal features from individual channels may be insufficient to comprehensively characterize the complex patterns of sleep stages.

**Figure 3:**
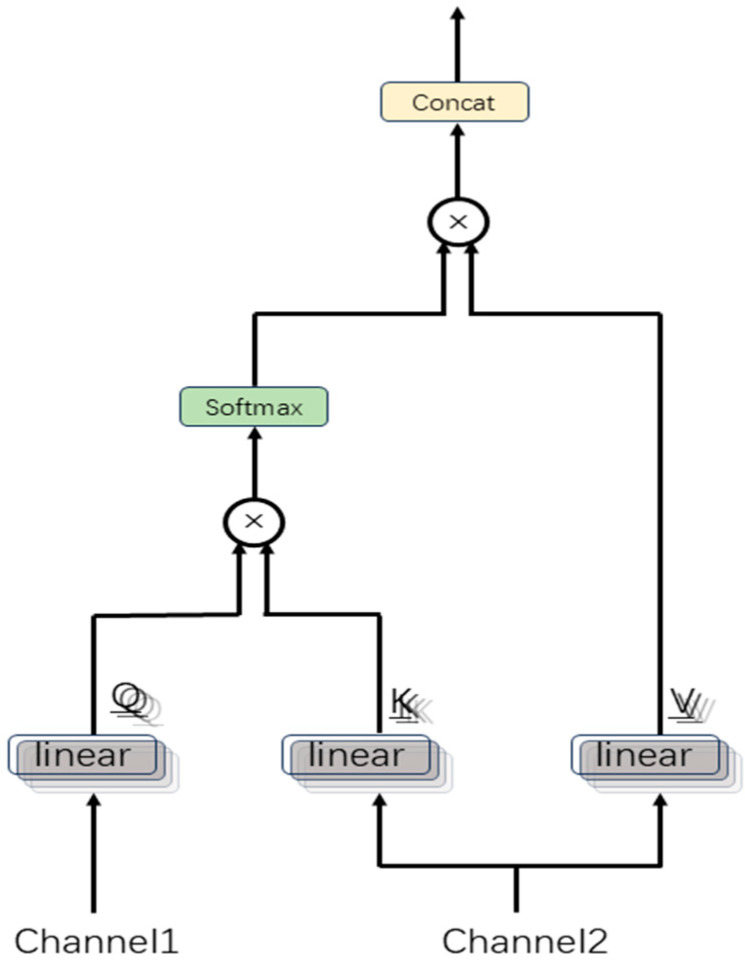
Architecture of multi-head attention between two channels.

To address this, we propose a MCAF module that dynamically fuses multi-channel features through a multi-head cross-attention mechanism, as illustrated in Figure 3.

The input tensor is projected into queries (Q), keys (K), and values (V) through linear layers, yielding the output $QKV\in R^{B\times C\times L\times 3D}$*,* which is then split along the last dimension into $Q,K,V\in R^{B\times C\times L\times D}$ (C  represents the number of input channels).

Conventional attention mechanisms are limited to capturing feature relationships within a single subspace, thereby restricting their representational capacity. In contrast, the multi-head attention mechanism addresses this limitation by partitioning the features into multiple subspaces (“heads”), where attention computations are performed independently in each subspace. This enables the model to learn complex interactions from multiple perspectives in parallel.

Specifically, for each channel, we divide Q, K, V into sub-dimensions according to the number of heads H, $Q,K,V\in R^{B\times C\times L\times H\times d_{h}},d_{h}=D/H$.

The multi-channel cross-attention mechanism is designed to model inter-channel dependencies among different physiological signal modalities. Specifically, the mechanism computes scaled dot products between queries (Q) from one channel and keys (K) from another channel to evaluate feature-level correlations across temporal positions.

These attention scores represent cross-channel attention distributions, quantifying how the representation of one channel at the current timestep attends to all temporal features of another channel.

Computing Attention Scores:

(1) $$\begin{array}{c} A_{h,i,j}=\frac{Q_{h,i}\cdot K_{h,j}^{T}}{\sqrt{D_{h}}}, \end{array}$$

where h denotes the attention head index, i, j represent channel indices, and $\sqrt{D_{h}}\;$ serves as the scaling factor for gradient stabilization.

Apply softmax normalization to the attention scores:

(2) $$\begin{array}{c} W_{h,i,j}=Softmax\left(A_{h,i,j},dim=-1\right). \end{array}$$

Compute the weighted features:

(3) $$\begin{array}{c} O_{h}=\sum_{j}W_{h,i,j}\cdot V_{h,j}. \end{array}$$

The outputs of all heads are concatenated and reshaped to form $O\in R^{B\times C\times L\times D}$. To further integrate the multi-head representations and enhance the feature expressiveness, a linear transformation is applied:

(4) $$\begin{array}{c} O^{\prime }=O\cdot W_{O}, \end{array}$$

where $W_{O}\in R^{D\times D}$ is a learnable parameter matrix.

**Figure 4:**
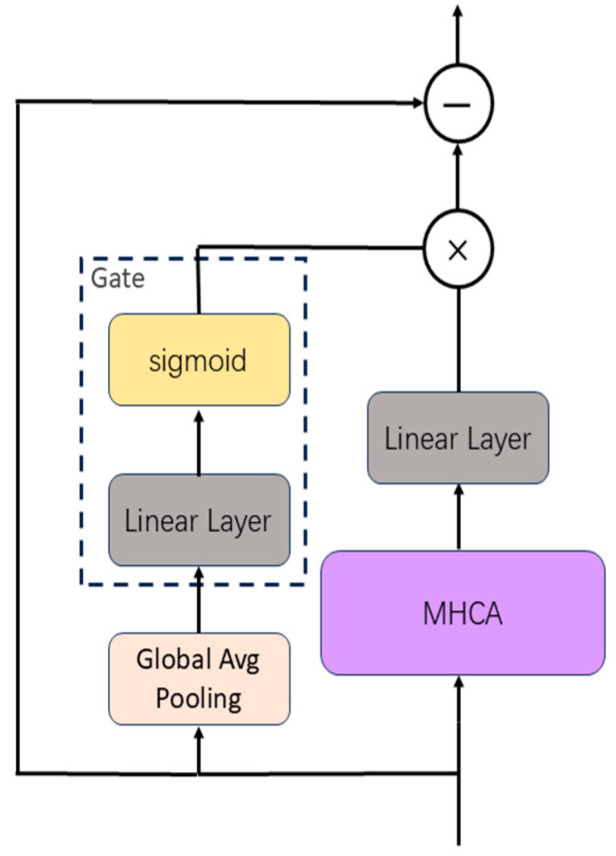
Illustration of the MCAF mechanism integrating gated multi-head cross-attention with residual connections.

Multi-channel physiological signals are characterized by high dimensionality, noise contamination, non-stationarity, and substantial inter-sample variability. Different signal patterns across samples may require varying degrees of attention and output contribution. To adaptively modulate the contribution of attention outputs, we propose a dynamic gating mechanism, as illustrated in Figure 4.

First, we compute the mean of the input $X\in R^{B\times C\times L\times D}$ along both channel and temporal dimensions to extract global features, compressing the multi-channel temporal information into a batch-wise feature vector that captures the global context of the input.

(5) $$\begin{array}{c} X_{mean}=\frac{1}{C\cdot L}\sum_{c=1}^{C}\sum_{l=1}^{L}X_{b,c,l}. \end{array}$$

The gating module (linear layer + sigmoid) generates scalar weights:

(6) $$\begin{array}{c} G=\sigma \left(X_{mean}\cdot W_{G}+b\right), \end{array}$$

where $G\in R^{B}$ represents sample-adaptive scaling factors that modulate how input features contribute to the fused output. $W_{G}\in R^{D\times 1}$ denotes the learnable weight matrix and b represents the bias term.

The gate weights are broadcast to $R^{B\times 1\times 1\times 1}$, then multiplied by the projected output $O^{\prime }$, and finally combined with input X via residual connection. The fused feature Y is expressed as:

(7) $$\begin{array}{c} Y=X+G\cdot O^{\prime }. \end{array}$$

The gating mechanism modulates the magnitude of fused features, while the residual connections ensure direct information propagation, effectively mitigating gradient vanishing. This gated residual refinement enhances the expressive power of feature representations, significantly improving the model’s capacity to capture complex sleep stage patterns.

### 2.4. Classification

First, the output $Y\in R^{B\times C\times D}$ of the multi-channel fusion block is flattened into $Y^{\prime }\in R^{B\times (C\times D)}$. The features are subsequently processed by a module comprising two fully connected layers, which integrate the representations derived from prior structures for sleep stage classification. The first fully connected layer employs a ReLU activation function, followed by a dropout layer to mitigate overfitting. A softmax function is then applied to produce probability distributions across mutually exclusive sleep stage classes. During model training, we employ the AdamW optimizer [23], which implements a more effective weight decay decoupling mechanism compared to standard Adam [24], thereby replacing conventional L2 regularization.

### 2.5. Experiments

#### 2.5.1. Dataset

To evaluate the performance of our proposed model, we conducted experiments on two publicly available datasets: SleepEDF-20 and SleepEDF-78. These are described in Table 1.

SleepEDF-20: This subset of the Sleep-EDF Expanded dataset (2013 version) [25,26] comprises polysomnography (PSG) recordings from 20 healthy subjects aged 25 to 34 years. Each subject was monitored for two consecutive nights, resulting in 39 whole-night PSG records, as the second night’s recording for subject 13 was lost due to equipment failure (e.g., cassette or laserdisc malfunction). Sleep experts manually annotated each 30 s epoch according to the Rechtschaffen and Kales (R&K) [27] criteria, classifying them into one of eight stages: Wake (W), Sleep Stages 1–4 (S1, S2, S3, S4), Rapid Eye Movement (REM), MOVEMENT, or UNKNOWN. Consistent with prior studies [15,19,22,28], we combined S3 and S4 into a single stage (N3) and excluded MOVEMENT and UNKNOWN epochs. For sleep stage classification, we utilized the Fpz-Cz EEG, Pz-Oz EEG, and ROC-LOC EOG (horizontal) channels, sampled at 100 Hz. Following established practice [16,22], only the 60 min time window centered around the in-bed period of each recording was analyzed.

SleepEDF-78: Derived from the expanded Sleep-EDF database (2018 version) [26,28], this dataset includes PSG recordings from 78 subjects aged 25 to 101 years, totaling 153 whole-night sleep records. Each subject was recorded for two nights, though equipment errors led to the loss of one record each for subjects 13, 36, and 52. Epochs of 30 s were manually scored using the same R&K standards as SleepEDF-20, with identical stage labels. As with SleepEDF-20, we merged S3 and S4 into N3 and removed MOVEMENT and UNKNOWN stages. The Fpz-Cz EEG, Pz-Oz EEG, and ROC-LOC EOG (horizontal) channels, sampled at 100 Hz, were used for sleep staging.

#### 2.5.2. Parameter

As described in Section 2.1, each 30 s epoch of polysomnography (PSG) signals was transformed into a time-frequency representation via a 256-point Short-Time Fourier Transform (STFT) with a 2 s Hamming window (50% overlap). The resulting spectra were converted to log-power spectra, producing a time-frequency matrix $X\in R^{T\times F}$, where F=128 frequency bins and T=29 time points. For each channel, the time-frequency matrix was standardized to zero mean and unit variance across all elements prior to being fed into the MCAF-Net model.

In the MCAF-Net architecture, the feature dimension D=128 was chosen to align with the input frequency bins. Each of the three PSG channels was processed by $N_{C}=3$ dedicated temporal convolution modules, each comprising a convolutional layer with a kernel size of 3, same padding, a depth-wise separable convolution with groups equal to D, and an output channel expansion factor of 2, followed by GELU activation. Channel-specific features were integrated through $N_{A}=2$ cross-channel attention layers, each equipped with H=4 parallel attention heads. A dropout rate of 0.5 was applied in the classification head, which comprised two fully connected layers: a hidden layer with $H_{F}=512$ units accepting an input of dimension 3·D, activated by ReLU, and an output layer with C=5 units corresponding to the five sleep stages (Wake, N1, N2, N3, REM).

The MCAF-Net model was implemented using PyTorch 2.4.1, with training conducted on an NVIDIA GeForce RTX 3050 Ti GPU(NVIDIA Corporation, Santa Clara, CA, USA). The model requires only 0.01 GFLOPs for a single forward pass, with a parameter count of 0.63M and a peak memory usage of 151.59 MB during inference. To evaluate the performance of MCAF-Net, we employed subject-wise k-fold cross-validation on the SleepEDF-20 and SleepEDF-78 datasets, dividing subjects into k groups with k values of 20 and 10, respectively, consistent with prior studies. During the training phase, we held out an independent validation set with the same number of subjects as the test set for model evaluation. The model was optimized using cross-entropy loss with the AdamW optimizer [23], configured with the following parameters: learning rate of $5\times 10^{-6}$, weight decay of $10^{-2}$, $\beta_{1}=0.9$, $\beta_{2}=0.999$, and $\varepsilon =10^{-2}$. We employed a mini-batch size of 64 and implemented an early stopping criterion that terminated training if the validation accuracy failed to improve for 20 consecutive epochs.

## 3. Experimental Results

### 3.1. Sleep Staging Performance

Table 2 and Table 3 present the confusion matrices of MCAF-Net on the Sleep-EDF-20 and Sleep-EDF-78 datasets, with rows and columns representing ground truth and predicted labels, respectively. The right side of these tables reports per-class evaluation metrics for MCAF-Net and baseline methods, including Precision (PR, the proportion of correctly classified instances among those predicted as a given class), Recall (RE, the proportion of correctly predicted instances among all true instances of a given class), and F1-score (F1, the harmonic mean of Precision and Recall, providing a balanced measure of classification performance). The best values for each metric are highlighted in bold. The high values along the diagonal of the confusion matrices indicate accurate classification for most epochs. Although the classification performance for the N1 stage is slightly lower compared to other stages, MCAF-Net demonstrates significant improvements over baseline models in this regard.

**Figure 5:**
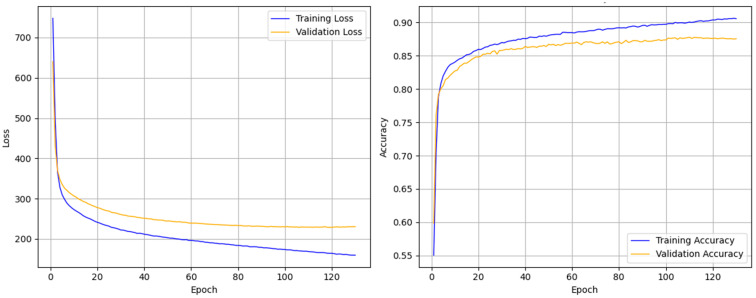
Accuracy and loss during training of multi-channel SleepNet for a randomly selected fold (i.e., fold 6) in the SleepEDF-20 dataset.

Figure 5 illustrates the loss and accuracy curves during the training process, demonstrating that the multi-channel SleepNet can rapidly converge and achieve stable performance after only a few training epochs. Throughout training, the accuracy on the training set continuously improves, while the loss steadily decreases. Meanwhile, both the accuracy and loss on the validation set stabilize after a few epochs, indicating that MCAF-Net exhibits strong robustness in mitigating model overfitting.

### 3.2. Hypnogram

**Figure 6:**
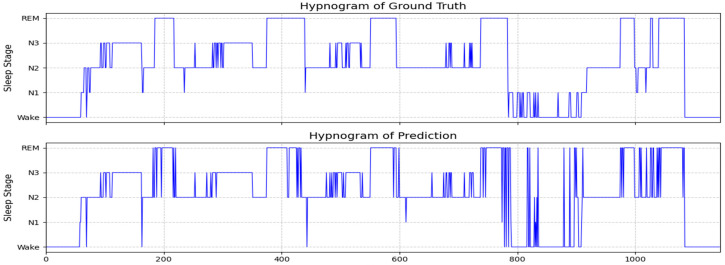
The ground truth sleep stages and MCAF-Net classification results are presented for subject SC4161E0 from the Sleep-EDF-20 dataset, with achieved performance metrics of 88.6% accuracy and 0.75 F1-score.

Figure 6 presents the hypnogram of subject SC4161E0 from the Sleep-EDF-20 dataset, displaying the ground truth annotations (upper panel) and corresponding predictions from the Multi-Channel Attention Fusion Network (MCAF-Net, lower panel). The predicted sleep stages demonstrate high concordance with the ground truth. For this subject, MCAF-Net achieved an accuracy of 88.6% and a macro-averaged F1-score of 0.75 in sleep stage classification. Error analysis reveals that most misclassifications occurred in the N1 stage, consistent with the results presented in Table 2.

Visually, the MCAF-Net-generated hypnogram exhibits slightly reduced temporal smoothness compared to the ground truth, with occasional abrupt stage transitions. This phenomenon primarily stems from our input processing strategy, where temporal sequences were shuffled during training, intentionally disregarding inter-epoch dependencies to reduce model complexity. Despite this architectural simplification, the proposed framework maintains superior classification performance while offering significant computational efficiency advantages, making it particularly suitable for practical clinical applications.

### 3.3. Performance Comparison

We compared the performance of MCAF-Net with existing methods. The overall performance was evaluated using accuracy, Cohen’s kappa (κ) [29], and macro-F1 (MF1) [30], while per-class performance was assessed via F1-score. As shown in Table 4, MCAF-Net achieves superior classification performance compared to other methods due to its effective single-channel feature extraction and efficient multi-channel feature fusion capabilities.

Compared with single-channel-based methods [16,17,19], MCAF-Net leverages multi-channel signals to fully exploit the contributions of different modalities for sleep staging. Unlike approaches that employ shared filters for multi-channel feature extraction [15], MCAF-Net accounts for the heterogeneity among different channel signals and independently extracts information from each channel. Furthermore, in contrast to methods utilizing other multi-channel fusion strategies [21,22], MCAF-Net adopts a dynamic gated cross-channel multi-head fusion mechanism to comprehensively model interactions between different signals. Consequently, the proposed MCAF-Net effectively utilizes multi-channel information and exploits their complementary relationships, enabling more robust and comprehensive sleep stage classification

### 3.4. Ablation Study on Multi-Channel Fusion

To evaluate the effectiveness of the MCAF module in feature fusion, we compared the original MCAF-Net with several ablated variants on the Sleep-EDF-20 dataset, where the Channel-Aware Attention module was removed while keeping all other parameters identical. The compared variants are described as follows:

1. Fpz-Cz EEG: A single-channel feature extraction block processing only the Fpz-Cz EEG channel.
2. Pz-Oz EEG: A single-channel feature extraction block processing only the Pz-Oz EEG channel.
3. EOG: A single-channel feature extraction block processing only the EOG channel.
4. Concatenation: The three channels were concatenated at the input stage without employing any multi-channel feature fusion block.

**Figure 7:**
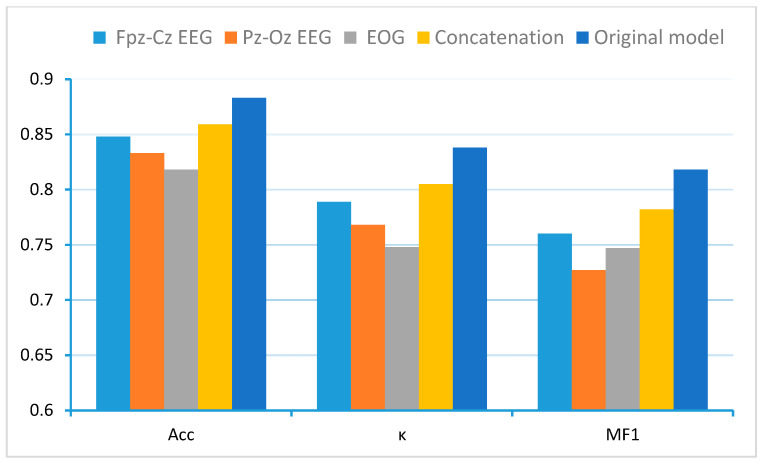
Performance comparison of MCAF-Net with single-channel baselines and naive concatenation on Sleep-EDF-20.

Figure 7 demonstrates the superior performance of MCAF-Net compared to its variant models. Based on variants 1–3, comparative analysis between the original MCAF-Net and single-channel models reveals that the multi-channel fusion-equipped architecture significantly outperforms single-channel counterparts lacking this module. This finding substantiates that appropriate fusion strategies can more comprehensively utilize sleep stage information embedded in different physiological signals. Furthermore, when compared with the naive concatenation approach (variant 4), our proposed Dynamic Gated Cross-channel Multi-head Attention Fusion (Channel-Aware Attention) module demonstrates significantly more effective integration of multi-channel information, consequently achieving superior classification performance. The results highlight that simply combining multiple channels without sophisticated feature interaction modeling is insufficient for optimal sleep stage classification.

### 3.5. Ablation Study on TemporalConv and Channel-Aware Attention

To further evaluate the contributions of the Channel-Aware Attention and TemporalConv modules in MCAF-Net, we conducted an ablation study on the Sleep-EDF-20 dataset, systematically removing each module while keeping all other parameters unchanged. The performance was assessed using accuracy (ACC), Cohen’s Kappa (κ), macro-averaged F1-score (MF1), sensitivity (Sens), and specificity (Spec). The results are presented in Table 5.

As shown in Table 5, removing the Channel-Aware Attention module results in a noticeable performance decline, with accuracy dropping from 88.3% to 87.4% and MF1 decreasing from 81.8% to 81.0%. This suggests that the Channel-Aware Attention module significantly enhances multi-channel feature fusion by modeling dynamic inter-channel interactions, thereby improving classification performance. Similarly, removing the TemporalConv module leads to a more substantial performance degradation, with accuracy decreasing to 86.0% and MF1 dropping to 78.6. This underscores the critical role of the TemporalConv module in extracting temporal features across channels, particularly in capturing dynamic patterns associated with sleep stages.

The complete model (TemporalConv + Channel-Aware Attention) outperforms both ablation variants across all metrics, demonstrating the synergistic effect of the two modules in significantly enhancing MCAF-Net’s performance. The improvements in sensitivity and specificity further indicate that the combination of these modules strengthens the model’s ability to classify both positive and negative samples effectively. These results validate the importance of Channel-Aware Attention and TemporalConv in MCAF-Net, highlighting their complementary roles in ensuring efficient feature extraction and fusion, ultimately leading to more robust sleep stage classification.

### 3.6. Analysis of Channel-Wise Attention Weights in MCAF-Net

**Figure 8:**
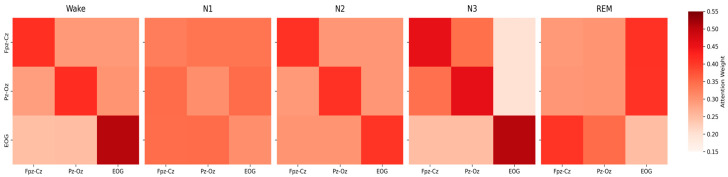
Channel-wise attention distribution across sleep stages.

To elucidate the MCAF-Net model’s ability to capture inter-channel dependencies across sleep stages, we analyzed the channel-wise attention weights derived from the cross-attention mechanism in the model. As shown in Figure 8, the attention weights, aggregated across four attention heads and averaged over the temporal dimension, are presented as 3 × 3 matrices for each sleep stage.

During wakefulness, high self-attention for Fpz-Cz and Pz-Oz reflects the model’s focus on alpha waves, while strong EOG attention captures frequent eye movements, consistent with the high F1-score in Table 2. Moderate cross-channel weights indicate effective EEG-EOG integration. In the N1 stage, attention weights are evenly distributed, reflecting its transitional nature, with subtle theta waves and slow eye movements. Slightly higher Pz-Oz to EOG weights align with Table 6, where their combination improved N1 classification. For N2, high self-attention across all channels highlights the model’s emphasis on sleep spindles, K-complexes, and minor eye movement features, consistent with robust F1-scores. In N3, dominant self-attention for Fpz-Cz and Pz-Oz reflects slow-wave activity, while low cross-channel weights to EOG indicate minimal reliance on eye signals, matching performance trends in Table 6. During REM, elevated cross-channel attention from EEG to EOG captures rapid eye movements, while moderate EOG to Pz-Oz weights reflect theta activity contributions, supporting the model’s strong REM classification.

The attention weight analysis reveals that MCAF-Net effectively captures sleep stage-specific physiological characteristics through its Channel-Aware Attention mechanism. The high self-attention weights in Wake and N3 stages correspond to distinct EEG features (alpha and slow waves, respectively), while the elevated EOG-related weights in REM underscore the importance of rapid eye movements. The uniform weights in N1 reflect its transitional nature, posing challenges for classification, as evidenced by the lower F1-score. These findings validate the model’s ability to adaptively prioritize channel interactions, aligning with the performance improvements observed in multi-channel settings. The attention mechanism’s focus on EOG in REM and Pz-Oz in N1 and REM highlights its sensitivity to physiologically relevant features, enhancing the interpretability and robustness of MCAF-Net for sleep stage classification.

## 4. Discussion

MCAF-Net achieves performance improvements by employing a dynamic gated cross-channel multi-head attention mechanism to effectively integrate multi-channel information. Although single-channel EEG approaches remain prevalent due to their potential applicability in longitudinal studies and homesleep monitoring [14,16,31,32,33], clinical sleep stage classification inherently requires the participation of multiple channels. Multi-modal signals significantly enhance staging accuracy, with their impact varying across different sleep stages. Figure 7 demonstrates the critical importance of cross-channel feature fusion.

Compared to the single-channel MCAF-Net configuration using only the Fpz-Cz EEG channel, the multi-channel model incorporating Fpz-Cz, Pz-Oz, and EOG achieves a 4.9% increase in accuracy and a 7.8% improvement in macro F1-score (MF1). To evaluate the contributions of individual channels to different sleep stages, we systematically tested various channel combinations as inputs to MCAF-Net. As shown in Table 4, the Fpz-Cz channel was selected as the baseline due to its superior performance compared to other single-channel configurations.

The classification results on the Sleep-EDF-78 dataset are presented in Table 4. The inclusion of the Pz-Oz channel alongside Fpz-Cz significantly improves model performance, particularly for the N1 and REM stages, with F1-score enhancements of 9.8% and 10.3%, respectively. The Pz-Oz channel, which captures EEG signals from the parieto-occipital region, is particularly sensitive to alpha and theta oscillations associated with N1 and REM sleep. The marked improvement in N1 classification suggests that Pz-Oz provides critical spectral information for detecting transitional light sleep. Similarly, gains in REM and N3 classification are attributed to Pz-Oz’s ability to supplement frontal EEG features (Fpz-Cz) with posterior slow-wave and rhythmic activity. These results demonstrate the advantage of dual-channel EEG configurations in capturing complementary neural signatures for improved staging accuracy.

The addition of EOG signals to Fpz-Cz further enhances performance, particularly for REM detection, where the F1-score increases from 70.6% to 83.7%. This improvement reflects EOG’s unique sensitivity to rapid eye movements, a hallmark of REM sleep. The N1 F1-score also rises by 9.7%, likely due to EOG’s detection of slow eye movements (SEMs) prevalent during this stage. In contrast, N3 classification remains stable, as deep sleep lacks prominent ocular activity. Notably, the Fpz-Cz + EOG combination outperforms Fpz-Cz + Pz-Oz in REM classification, underscoring EOG’s discriminative power for phasic sleep events. Both configurations yield comparable N2 scores (~87%), suggesting similar contributions to spindle detection. However, their synergistic integration in the three-channel setup further refines N2 classification (87.2%), highlighting complementary feature interactions.

The optimal performance is achieved by combining Fpz-Cz, Pz-Oz, and EOG, with the N1 F1-score reaching 52.1%—a 14.8% absolute improvement over the single-channel baseline. REM classification also benefits from integrated ocular and posterior EEG features, attaining an F1-score of 83.7%. This tri-modal configuration leverages the strengths of each signal: Fpz-Cz and Pz-Oz provide spatially distinct EEG coverage (frontal vs. parieto-occipital), while EOG captures auxiliary oculomotor dynamics critical for phasic sleep stages. These findings align with sleep neurophysiology, demonstrating that MCAF-Net effectively fuses multi-modal features, mirroring clinical polysomnography practices where experts synthesize EEG, EOG, and EMG data for staging.

This study primarily focuses on addressing the challenge of multi-channel physiological signal feature fusion by proposing a lightweight cross-channel attention mechanism to improve sleep stage classification performance. To ensure fair and domain-relevant evaluation, we compared our method with widely used and representative baseline models in the sleep staging field. However, comparisons with emerging neural network topological variants such as Kolmogorov–Arnold Networks (KANs) were not included. Given the promising potential of KAN and similar variants in function approximation and model interpretability, future work will consider integrating these methods into our framework for more comprehensive and in-depth comparative studies to further validate the model’s effectiveness and applicability.

Although the three-channel configuration achieved the best overall performance, the F1-score for the N1 stage remained lower than other stages, reflecting the intrinsic ambiguity of its signal features. Furthermore, the Sleep-EDF-20 and Sleep-EDF-78 datasets used in this study contain only 20 and 78 subjects, respectively, representing a relatively small sample size that may limit the generalizability of the model evaluation. Additionally, these datasets include only EEG and EOG signals, without other polysomnography (PSG) modalities such as electromyography (EMG) or respiratory data. Prior studies suggest that incorporating such signals could further enhance classification performance [34]. Moreover, integrating high-density EEG montages commonly used in sleep research [35] may improve model robustness and accuracy. Future research should explore additional channel combinations, expand the sample size, and include more diverse clinical populations to overcome current limitations and validate the method’s generalizability in real-world scenarios.

## 5. Conclusions

In this paper, we propose MCAF-Net, a novel deep learning model for sleep stage classification. The core components of MCAF-Net are the TemporalConv module and the Dynamic Gated Multi-head Cross-channel Attention Fusion (MCAF) mechanism. The TemporalConv module is designed to extract channel-specific features from individual physiological signals, while the MCAF mechanism effectively captures and integrates cross-channel correlations to enhance multi-channel data fusion. The proposed approach was evaluated on two widely used datasets, Sleep-EDF-20 and Sleep-EDF-78, demonstrating superior performance in sleep stage classification compared to existing methods. Furthermore, ablation studies were conducted to validate the effectiveness and robustness of the proposed model.

In future work, given that the multi-head attention mechanism is a pivotal component of MCAF-Net, we aim to optimize its computational efficiency to further enhance the model’s performance. As our model adopts a one-to-one architecture, processing each epoch independently without leveraging contextual information, certain samples in the model’s output exhibit anomalous stage transitions. These limitations will be addressed in future research to improve the model’s temporal coherence and classification accuracy.

## Figures and Tables

**Table 1:** Details of employed datasets (each data sample is a 30 s epoch).

Dateset	Subjects	W	N1	N2	N3	REM	Total Sample
SleepEDF-20	20	9118	2804	17,799	5703	77,177	43,141
		21.10%	6.50%	41.30%	13.20%	17.90%	
SleepEDF-78	79	66,822	21,522	69,132	13,039	25,835	196,350
		14.30%	3.20%	43.70%	18.50%	20.30%	

**Table 2:** Performance metrics and confusion matrix of the SleepEDF-20 dataset.

	Predicted					MCAF-Net			MultiChannelNet [22]			AttnSleep [19]		
	W	N1	N2	N3	REM	PR	RE	F1	PR	RE	F1	PR	RE	F1
W	**8652**	224	98	15	150	92.8	**94.7**	**93.7**	**93.4**	92.1	92.8	89.6	89.7	89.7
N1	454	**1096**	657	5	610	**65.2**	38.8	48.7	55.0	**44.4**	**49.1**	47.1	39.1	42.8
N2	106	206	**16,525**	411	673	**89.6**	**92.2**	**90.9**	89.5	90.3	90.0	89.1	88.6	88.8
N3	17	0	646	**4768**	4	**91.7**	87.7	89.6	89.1	89.5	89.3	80.7	**89.8**	**90.2**
REM	91	154	512	3	**6955**	**82.9**	**90.1**	**86.4**	82.3	87.5	84.8	76.1	82.2	79.0

**Table 3:** Performance metrics and confusion matrix of the SleepEDF-78 dataset.

	Predicted					MCAF-Net			MultiChannelNet [22]			AttnSleep [19]		
	W	N1	N2	N3	REM	PR	RE	F1	PR	RE	F1	PR	RE	F1
W	**57,878**	2121	346	32	370	94.0	**95.3**	**94.6**	**95.0**	93.1	94.0	92.3	91.8	92.0
N1	2902	**9061**	5713	36	1854	**59.7**	46.3	**52.1**	58.1	**48.7**	53.0	45.3	39.2	42.1
N2	412	2682	**56,410**	1265	2078	**84.7**	89.8	**87.2**	84.0	**90.0**	86.9	83.5	86.5	85
N3	44	11	2326	**9457**	16	**87.6**	79.8	**83.5**	83.1	80.7	81.8	82.3	**82.0**	82.1
REM	343	1312	1805	5	**20,021**	**82.3**	**85.2**	**83.7**	82.0	83.1	82.6	73.1	75.3	74.2

**Table 4:** Performance comparison with previous methods on two datasets.

		Overall Metrics			Per-Class F1_Score				
Dataset	System	Acc	κ	MF1	W	N1	N2	N3	REM
SleepEDF-20	MCAF-Net	**88.3**	**0.84**	**81.8**	**93.7**	48.7	**90.9**	89.6	**86.4**
	MultiChannelSleepNet [22]	87.2	0.82	81.2	92.8	**49.1**	90.0	89.3	84.8
	SeqSleepNet [15]	86.0	0.81	79.7	-	-	-	-	-
	AttnSleep [19]	84.4	0.79	78.1	89.7	42.8	88.8	**90.2**	79.0
	DeepSleepNet [16]	82.0	0.76	76.9	-	-	-	-	-
	SleepEEGNet [17]	84.3	0.79	79.7	89.2	52.2	89.8	85.1	85.0
SleepEDF-78	MCAF-Net	**85.6**	**80.0**	**80.1**	**94.6**	52.1	**87.2**	**83.5**	**83.7**
	MultiChannelSleepNet [22]	85.0	0.79	79.6	94.0	**53.0**	86.9	81.8	82.6
	SeqSleepNet [15]	83.8	0.78	78.2	-	-	-	-	-
	AttnSleep [19]	81.3	0.74	75.3	92.0	42.1	85.0	82.1	74.2
	SleepTransformer [20]	81.4	0.74	74.3	91.7	40.4	84.3	77.9	77.2
	SleepEEGNet [17]	80.0	0.73	73.6	-	-	-	-	-

**Table 5:** Ablation study results for Channel-Aware Attention and TemporalConv.

Model Configuration	ACC	κ	MF1	Sens	Spec
TemporalConv	87.4	0.83	81.0	0.80	0.96
Channel-Aware Attention	86.0	0.80	78.6	0.77	0.96
TemporalConv + Channel-Aware Attention	88.3	0.84	81.8	0.81	0.97

**Table 6:** Performance comparison of multi-channel SleepNet using different input channels on the SleepEDF-78 dataset.

Input Channel	Overall Metrics			Per-Class F1-Score				
	Acc	κ	MF1	W	N1	N2	N3	REM
Fpz-cz	80.7	0.73	72.4	91.6	37.3	83.7	78.7	70.6
Fpz-cz + Pz-Oz	84.9	0.79	78.7	94.8	47.1	87.0	**83.6**	80.9
Fpz-cz + EOG	85.0	0.79	78.9	94.0	47.0	86.8	83.1	83.7
Fpz-cz + Pz-Oz + EOG	**85.6**	**80.0**	**80.1**	**94.6**	**52.1**	**87.2**	83.5	**83.7**

## Data Availability

The original contributions presented in this study are included in the article. Further inquiries can be directed to the corresponding author.
